# Profiling of repetitive RNA sequences in the blood plasma of patients with cancer

**DOI:** 10.1038/s41551-023-01081-7

**Published:** 2023-08-31

**Authors:** Roman E. Reggiardo, Sreelakshmi Velandi Maroli, Vikas Peddu, Andrew E. Davidson, Alexander Hill, Erin LaMontagne, Yassmin Al Aaraj, Miten Jain, Stephen Y. Chan, Daniel H. Kim

**Affiliations:** 1 Department of Biomolecular Engineering, University of California Santa Cruz, Santa Cruz, CA USA; 2 Department of Molecular, Cell and Developmental Biology, University of California Santa Cruz, Santa Cruz, CA USA; 3 Center for Pulmonary Vascular Biology and Medicine, Pittsburgh Heart, Lung, Blood Vascular Medicine Institute, Division of Cardiology, Department of Medicine, University of Pittsburgh School of Medicine, Pittsburgh, PA USA; 4 Institute for the Biology of Stem Cells, University of California Santa Cruz, Santa Cruz, CA USA; 5 Genomics Institute, University of California Santa Cruz, Santa Cruz, CA USA; 6 Center for Molecular Biology of RNA, University of California Santa Cruz, Santa Cruz, CA USA; 7 Canary Center at Stanford for Cancer Early Detection, Stanford University School of Medicine, Palo Alto, CA USA; 8 Present Address: Department of Bioengineering, Northeastern University, Boston, MA USA; 9 Present Address: Department of Physics, Northeastern University, Boston, MA USA

**Keywords:** Transcriptomics, Non-coding RNAs, Computational biology and bioinformatics, Biomarkers, Tumour biomarkers

## Abstract

Liquid biopsies provide a means for the profiling of cell-free RNAs secreted by cells throughout the body. Although well-annotated coding and non-coding transcripts in blood are readily detectable and can serve as biomarkers of disease, the overall diagnostic utility of the cell-free transcriptome remains unclear. Here we show that RNAs derived from transposable elements and other repeat elements are enriched in the cell-free transcriptome of patients with cancer, and that they serve as signatures for the accurate classification of the disease. We used repeat-element-aware liquid-biopsy technology and single-molecule nanopore sequencing to profile the cell-free transcriptome in plasma from patients with cancer and to examine millions of genomic features comprising all annotated genes and repeat elements throughout the genome. By aggregating individual repeat elements to the subfamily level, we found that samples with pancreatic cancer are enriched with specific Alu subfamilies, whereas other cancers have their own characteristic cell-free RNA profile. Our findings show that repetitive RNA sequences are abundant in blood and can be used as disease-specific diagnostic biomarkers.

## Abstract (web summary)

RNAs derived from transposable elements and other repeat elements are enriched in the cell-free transcriptome of patients with cancer and can serve as diagnostic signatures.

## Main

Of the 3 billion base pairs in the human genome, approximately 75% are transcribed into RNA^1^. The vast majority of these RNAs are not translated into proteins and are thus considered non-coding RNAs. Although non-coding RNAs such as microRNAs^2,3^ and long non-coding RNAs^4,5^ (lncRNAs) are well annotated, many other non-coding RNAs are generated throughout the genome, including RNAs transcribed from repeat elements such as transposable elements (TEs)^6^. There are over 5 million repeat element insertions in the human genome, with repeat sequences comprising roughly half the genomic sequence content^7^. TE RNAs in particular are aberrantly expressed in diseases such as cancer^8–13^, highlighting their potential as abundant and specific biomarkers of disease^14,15^.

Cell-free RNAs^16^ are released from cells that comprise the various tissues and organ systems throughout the human body^17,18^. The diagnostic and prognostic potential of cell-free RNA is evidenced by the prediction of pre-eclampsia in pregnancy^19–21^, and cell-free RNAs serve as biomarkers of diseases such as cancer^22–26^ and Alzheimer’s disease^27–29^. Cell-free RNAs have been profiled predominantly via whole-exome RNA sequencing (RNA-seq), which precludes the detection of repeat-derived and other non-coding RNA, or ribosomal-RNA-depleted total RNA-seq^30^. Studies using total RNA-seq for cell-free RNA have identified many well-annotated non-coding RNAs in human plasma, and a small fraction of repeat-derived cell-free RNAs (1–2%) in healthy individuals^31^. However, the diagnostic potential of the repeat-derived cell-free RNA transcriptome in the context of disease remains unknown.

Here we report that repeat-aware profiling of the cell-free RNA transcriptome (COMPLETE-seq) enables the in-depth characterization of disease-specific, repeat-derived cell-free RNAs, and the accurate classification of patients with cancer by leveraging the rich feature space of the repeat-derived cell-free RNA transcriptome. In marked contrast to the cell-free RNA transcriptomes of healthy individuals, patients with cancer show strong enrichment of TE- and other repeat-derived cell-free RNAs in their blood, with patients with pancreatic cancer showing high levels of short interspersed nuclear element (SINE)-derived cell-free RNAs from various Alu subfamily elements. We further show the generalizability of COMPLETE-seq by showing that repeat-aware classification of liver, lung, oesophageal, colorectal and stomach cancer cell-free RNA data^32^ shows improved performance compared with repeat-naive classifiers. Taken together, our results show that repeat-aware COMPLETE-seq profiling of the cell-free RNA transcriptome identifies a robust and dynamic repeat-element-derived RNA signature for the diagnosis of diseases such as cancer.

## Results

### COMPLETE-seq enables repeat-aware profiling of the cell-free RNA transcriptome

**Fig. 1:**
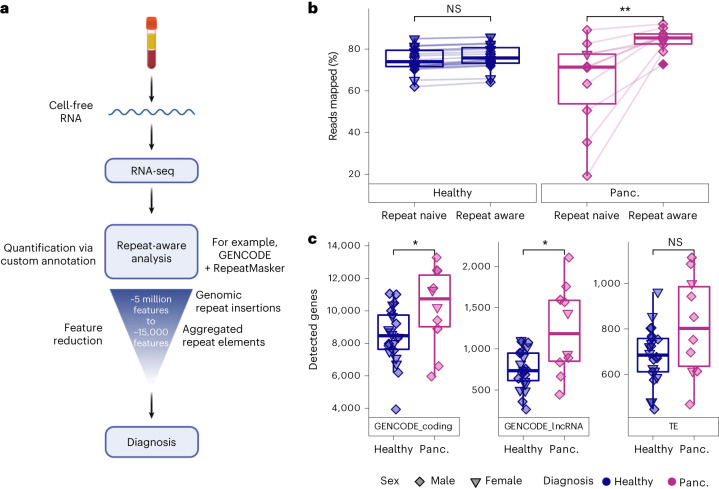
Cell-free RNA transcriptome profiling using repeat-aware COMPLETE-seq. **a**, Diagram of COMPLETE-seq RNA liquid-biopsy technology, highlighting the use of repeat-derived cell-free RNAs aggregated into a tractable feature set to enable diagnostic modelling. Created with BioRender.com. **b**, Comparison of mapping rates between use of a repeat-naive (GENCODE v.39) reference annotation (**P = 0.0039) and repeat-aware reference annotation (Wilcoxon, paired, two-sided). **c**, Comparison of gene detection distributions for each cohort across coding genes (GENCODE_coding; *P = 0.043), lncRNAs (GENCODE_lncRNA; *P = 0.035) and TE subfamilies (Wilcoxon, two-sided). For the box plots, the centre line represents the median, the box limits are upper and lower quartiles and whiskers represent 1.5× interquartile range. NS, not significant; panc., pancreatic cancer.

We developed the COMPLETE-seq technology to enable repeat-aware characterization of the cell-free RNA transcriptome. To generate cell-free RNA-seq data from human plasma, we leveraged a highly sensitive RNA-seq protocol that robustly detects both coding and non-coding RNAs^5^. Given that the human genome contains millions of repeat element insertions that have not been examined in the context of cell-free RNA, we created a custom transcriptome annotation for cell-free RNA quantification that incorporates both well-annotated coding and non-coding RNAs (that is, GENCODE) and over 5 million repeat element insertions found in the human genome (that is, RepeatMasker). We then aggregated the RNA signal from individual repeat element insertions to the subfamily element level^33^, reducing the number of repeat features from over 5 million to approximately 15,000 repeat features for disease classification and other downstream analyses (Fig. 1a).

**Extended Data Fig. 1:**
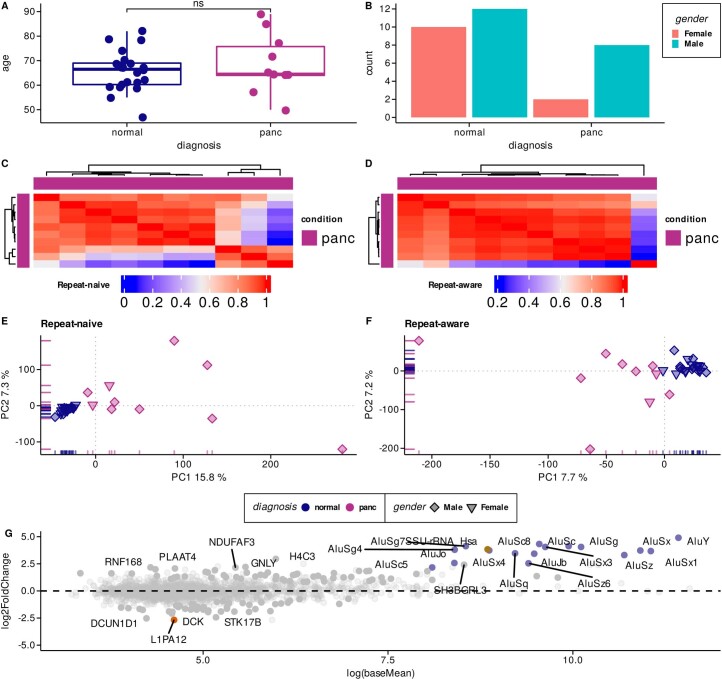
Performance overview of COMPLETE-seq on the internal cohort. **a**, Comparison of age distributions between cohorts (Wilcoxon, two-sided, ns: p > 0.05) enter line, median; box limits, upper and lower quartiles; whiskers, 1.5x interquartile range. **b**, Number of samples, stratified by gender, in each cohort. **c**, Heatmap (K-means) of Pearson correlation between panc samples using Repeat-naïve quantification. **d**, Heatmap (K-means) of Pearson correlation between panc samples using Repeat-aware quantification. **e**, PCA dimensions 1 & 2 calculated using variance-stabilized, Repeat-naive quantifications for normal and panc samples. **f**, PCA dimensions 1 & 2 calculated using variance-stabilized, Repeat-aware quantifications for normal and panc samples. **g**, MA plot of log2FoldChange between panc and normal samples compared to log-scale baseMean derived from DESeq2. Significantly DE genes/subfamilies are full opacity and colour.

Compared with using only well-annotated GENCODE coding and non-coding genes (repeat naive) for cell-free RNA quantification, the application of our COMPLETE-seq technology significantly enhanced the percentage of mapped reads in our cell-free RNA data from patients with pancreatic cancer (Fig. 1b and Extended Data Fig. 1). For healthy control cell-free RNA, however, the mapping rate difference between repeat-naive versus our repeat-aware approach was negligible (Fig. 1b). Notably, there were no significant differences between the total number of repeat subfamilies that were represented in the cell-free RNA of patients with pancreatic cancer and healthy individuals (Fig. 1c).

Both repeat-naive and repeat-aware quantification of the cell-free RNA transcriptomes of patients with pancreatic cancer and healthy individuals enabled robust, unsupervised disease identification in low-dimensional space via principal component analysis (Extended Data Fig. 1e,f). Compared with using only well-annotated GENCODE coding and non-coding genes for cell-free RNA quantification, however, the application of our repeat-aware technology increased the sample-to-sample correlation (Pearson) of cell-free RNA data from patients with pancreatic cancer (Extended Data Fig. 1c,d), indicating greater overall similarity when examining a more robust annotation of their cell-free RNA transcriptomes.

### TE RNAs and other repeat element RNAs are enriched in pancreatic cancer cell-free RNA

**Fig. 2:**
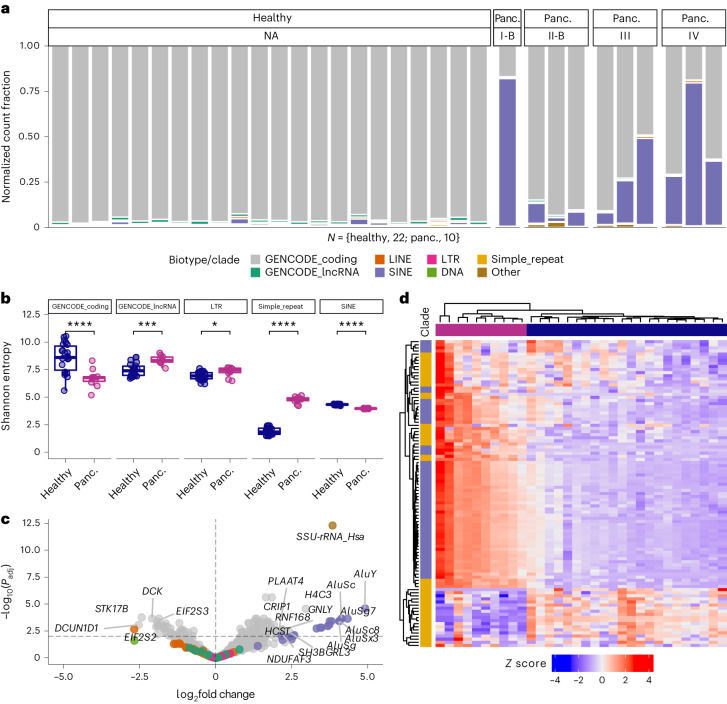
Disease-specific enrichment of repeat-derived cell-free RNA. **a**, Distribution of biotype representation (by DESeq2-normalized count) in cell-free RNA-seq quantifications for samples from each cohort, coloured by GENCODE biotype or repeat subfamily, and facetted by stage (NA for healthy samples). **b**, Comparison of significantly different (Wilcoxon, two-sided) Shannon entropy distributions for GENCODE biotype (****P = 9.6 × 10^−5^, ***P = 0.00019) and repeat subfamilies (*P = 0.014, ****P = 3.1 × 10^−8^). **c**, Volcano plot of significantly (*P* < 0.01) differentially expressed genes or repeat subfamilies derived from repeat-aware quantification, with horizontal and vertical lines drawn at −log_10_(0.01) and 0, respectively. **d**, Heat map (*K* means) of *Z* scores calculated from DESeq2-normalized counts of SINEs and simple repeats, with an average of at least five counts per sample across the dataset. For the box plots, the centre line represents the median, the box limits are upper and lower quartiles and whiskers represent 1.5× interquartile range. NA, not applicable.

To determine the repeat composition of cell-free RNA, we first examined repeat content at the superfamily level. In healthy individuals, less than 10% of the DESeq2-normalized cell-free RNA counts consistently corresponded to repeats. However, we found substantially larger fractions of repeat-derived cell-free RNAs across almost all patients with pancreatic cancer, with most of these cell-free RNAs being derived from SINE elements (Fig. 2a). We also found significant differences in the information content, as quantified by Shannon entropy^34^, of protein-coding RNAs, lncRNAs, and long terminal repeat (LTR), SINE and simple repeat superfamilies (Fig. 2b). These differences in biotype or repeat superfamily transcriptome diversity suggest dynamic changes in both the abundance and identity of cell-free RNAs in the context of diseases such as cancer.

**Extended Data Fig. 2:**
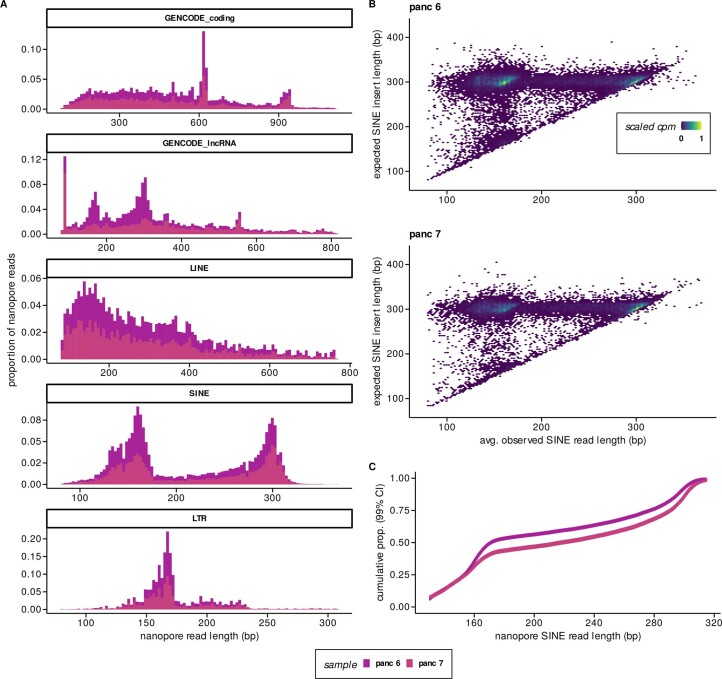
Nanopore sequencing of cell-free RNA reveals biotype-specific fragment-size patterns. **a**, Distribution of cell-free RNA lengths in base pairs (bp) for GENCODE biotypes or - repeat superfamily elements in pancreatic (panc 6, panc 7) cancer patients. **b**, Density plots depicting the relationship between expected (genomic SINE locus length) and observed SINE cell-free RNA length in pancreatic (panc 6, panc 7) cancer patients. **c**, Cumulative distribution function plot of SINE cell-free RNA length empirically calculated in pancreatic (panc 6, panc 7) cancer patients.

**Extended Data Fig. 3:**
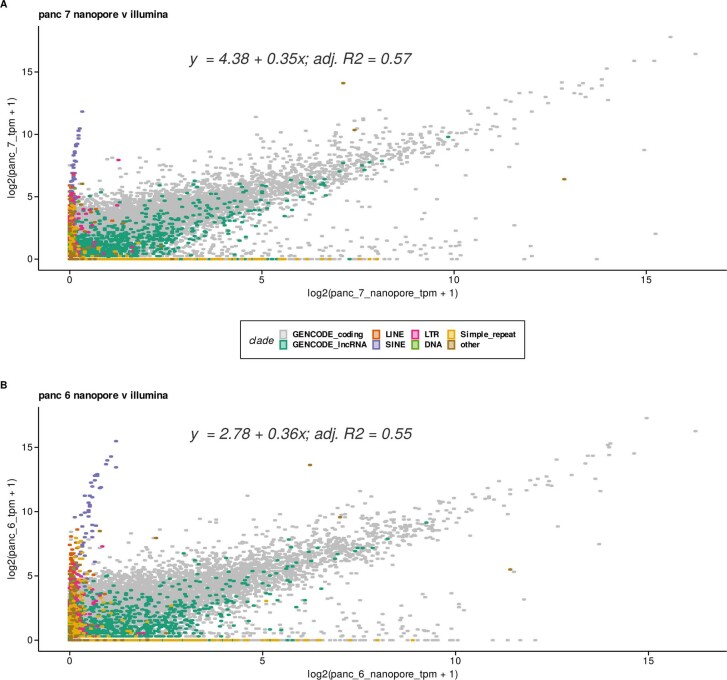
Nanopore and Illumina show agreement in the quantification of most GENCODE-annotated genes. **a**, Scatter plot depicting transcripts-per-million abundance for transcripts detected in matched nanopore and Illumina libraries from sample panc 7. Linear fit described. **b**, Scatter plot depicting transcripts-per-million abundance for transcripts detected in matched nanopore and Illumina libraries from sample panc 6. Linear fit described.

To further characterize the features of repeat-derived cell-free RNAs, we used full-length complementary DNA to perform single-molecule sequencing using nanopore technology^35^ on cell-free RNA samples from patients with pancreatic cancer that were also sequenced using Illumina technology. We examined the size distributions of protein-coding RNA, lncRNA, and long interspersed nuclear element (LINE)-, SINE- and LTR-derived cell-free RNAs from patients with pancreatic cancer, which revealed cell-free RNA transcripts up to 1,337 nt in length for protein-coding RNA (median 456 nt), 970 nt for lncRNA (median 303 nt), 1,002 nt for LINE (median 258 nt), 368 nt for SINE (median 185 nt) and 477 nt for LTR (median 167 nt) (Extended Data Fig. 2a). For SINE-derived cell-free RNA, we observed a bimodal length distribution, reflecting both full-length, ~300-nt-long Alu-derived RNA, along with a shorter species of Alu-derived RNA (Extended Data Fig. 2a). We then compared the expected length of SINE-derived RNA based on genomic alignment with the observed length of cell-free SINE RNA and found both full-length transcripts of expected size, and half-length cell-free SINE RNAs (Extended Data Fig. 2b,c). In addition, we compared the cell-free RNA abundances of all COMPLETE-seq-annotated genes and repeat subfamilies in matched nanopore and Illumina libraries, which revealed strong concordance between well-annotated coding and non-coding genes, with a bias towards Illumina for TE RNAs and towards nanopore for some simple repeat RNAs (Extended Data Fig. 3).

We next performed differential expression analysis and found that Alu subfamily elements were the most enriched TE signal in cell-free RNA from patients with pancreatic cancer, with AluY, AluSc, AluSg7, AluSc8, AluSx3 and AluSg subfamily elements among the most significantly enriched (*P* < 0.01) in patients with pancreatic cancer compared with healthy individuals (Fig. 2c). Further analysis of the significantly enriched repeat subfamilies showed that the upregulated Alu elements in pancreatic cancer cell-free RNA were highly abundant (Extended Data Fig. 1g), despite the lack of increase in overall SINE complexity in the pancreatic cancer cell-free RNA transcriptome (Fig. 2b). Capturing both the robust enrichment of Alu elements and the significant increase in simple repeat entropy in the pancreatic cancer cell-free RNA transcriptome, hierarchical clustering achieved perfect clustering of patients with pancreatic cancer (Fig. 2d). These repeat-aware analyses further contextualized the differences in the respective repeat superfamilies, where SINE-derived cell-free RNAs were uniform in their enrichment in our cohort of patients with pancreatic cancer, whereas simple repeat-derived cell-free RNAs were far more divergent, with some simple repeat-derived cell-free RNAs being enriched in healthy individuals (Fig. 2d).

### COMPLETE-seq reveals cancer-specific repeat element RNA signatures in cell-free RNA

**Extended Data Fig. 4:**
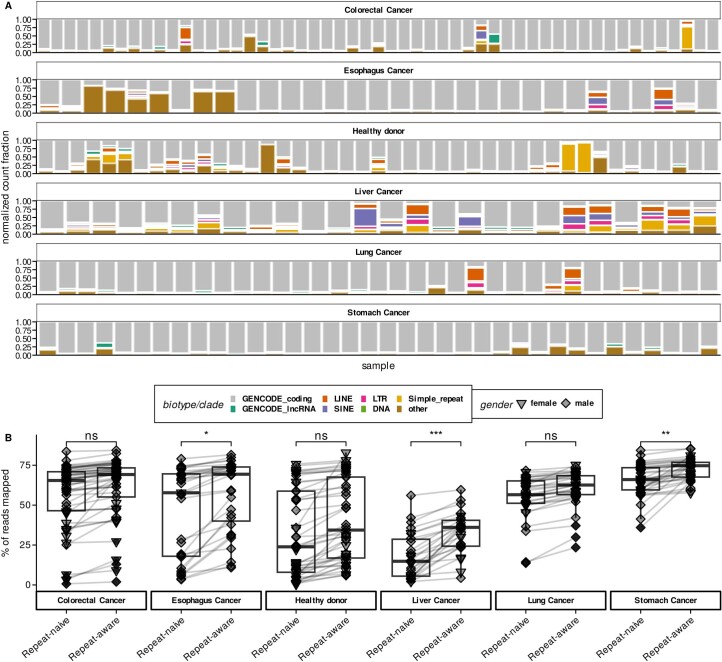
Repeat-aware analysis of cell-free RNA from 5 different cancers. **a**, Distribution of biotype representation (by DESeq2 normalized count) in cell-free RNA-seq quantifications for each cancer type, coloured by GENCODE biotype or repeat subfamily, and facetted by stage. **b**, Comparison of mapping rates between use of a Repeat-naïve (GENCODE v39) reference annotation and Repeat-aware reference annotation (Wilcoxon, paired, two-sided). center line, median; box limits, upper and lower quartiles; whiskers, 1.5x interquartile range. ns: p > 0.05, *: p <= 0.05, **: p <= 0.01, ***: p <= 0.001.

**Extended Data Fig. 5:**
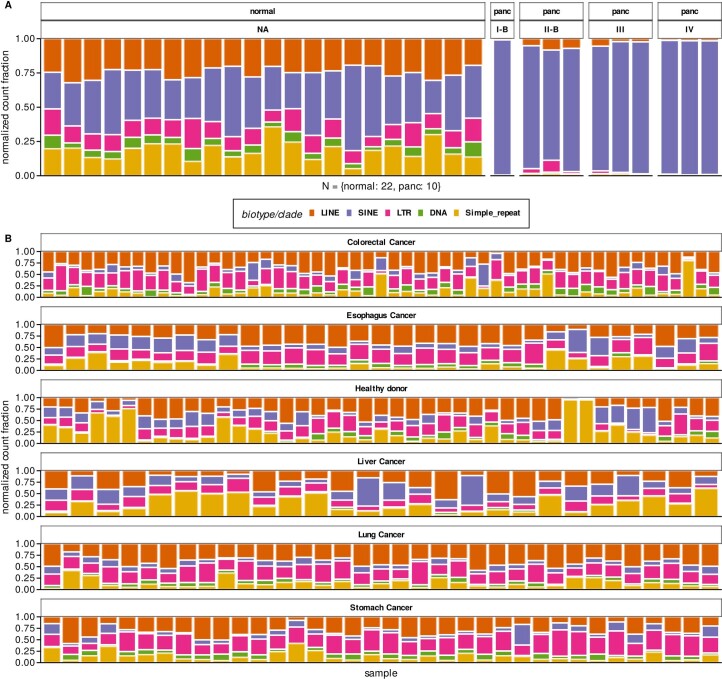
Repeat-specific diversity across internal and external cohorts. **a**, Distribution of repeat representation (by DESeq2 normalized count) in cell-free RNA-seq quantifications for pancreatic cancer, coloured by repeat subfamily, and facetted by stage. **b**, Distribution of repeat representation (by DESeq2 normalized count) in cell-free RNA-seq quantifications for each cancer type, colored by repeat subfamily.

To show the generalizability and applicability of COMPLETE-seq technology in enabling RNA liquid biopsy for cancer diagnosis, we used COMPLETE-seq quantification to analyse lung, liver, oesophageal, colorectal and stomach cancer cell-free RNA-seq data, along with their corresponding healthy controls^32^. We observed repeat superfamily variability in both healthy and cancer cell-free RNA transcriptomes (Extended Data Fig. 4 and Extended Data Fig. 5) and a significant (*P* < 0.05) increase in mapping rate by using our repeat-aware COMPLETE-seq annotation for analysing oesophageal, liver and stomach cancer cell-free RNA transcriptomes (Extended Data Fig. 4b).

**Fig. 3:**
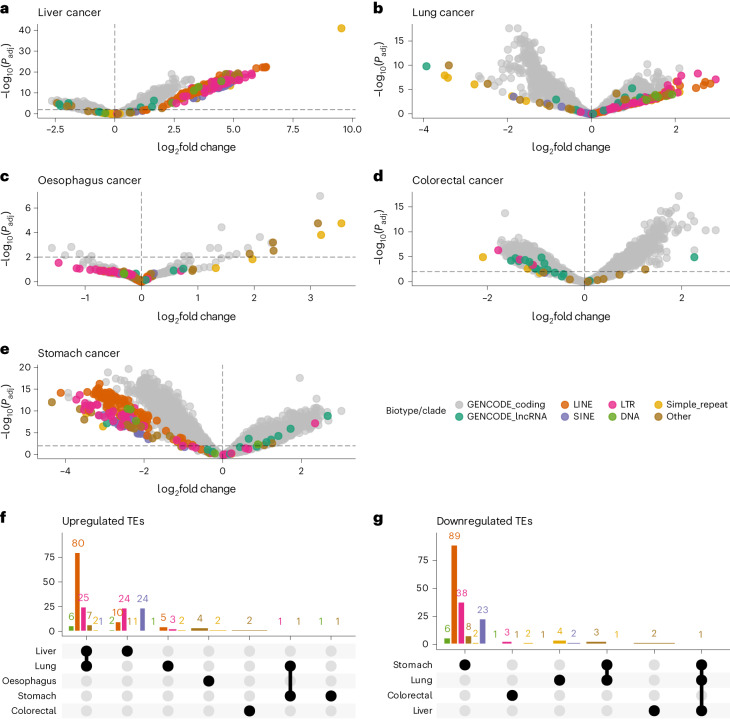
Disease-specific repeat-derived cell-free RNA signatures. **a**–**e**, Volcano plots of differentially expressed genes and repeat subfamilies derived from repeat-aware quantification of cell-free RNA-seq data for liver (**a**), lung (**b**), oesophagus (**c**), colorectal (**d**) and stomach (**e**) cancer. Horizontal and vertical lines drawn at −log_10_(0.01) and 0, respectively. **f**,**g**, UpSet plots showing the number of shared and unique upregulated (**f**) or downregulated (**g**) TE subfamilies across the different cancer types.

**Extended Data Fig. 6:**
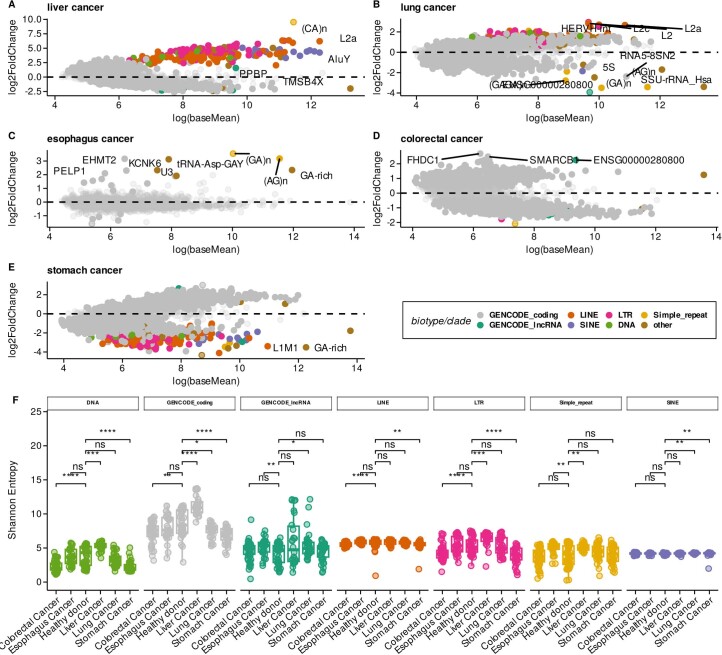
Differential expression and variability of repeat-elements in 5 different cancers. **a-e**, MA plots of log2FoldChange between labeled cancer type and healthy donor samples compared to log-scale baseMean derived from DESeq2. Significantly DE genes/repeat subfamilies are full opacity and color. **f**, Comparison of significantly different (Wilcoxon, two-sided) Shannon Entropy distributions for GENCODE biotypes and repeat subfamilies. center line, median; box limits, upper and lower quartiles; whiskers, 1.5x interquartile range. ns: p > 0.05, *: p <= 0.05, **: p <= 0.01, ***: p <= 0.001, ****: p <= 0.0001.

Performing pairwise comparisons between five different cancers and healthy individuals captured robust and significant (*P* < 0.01) differential expression of repeat-derived cell-free RNAs that were characteristic to each cancer type (Fig. 3a–e). Additional analyses of the significantly differentially expressed repeat subfamilies showed that these repeat-derived cell-free RNAs were also highly abundant (Extended Data Fig. 6), with significant changes to biotype or repeat superfamily entropy (*P* < 0.05) (Extended Data Fig. 6f). By comparing the significantly differentially expressed repeat RNA signal across cancer types, we identified cancer-specific TE- and other repeat-derived cell-free RNA enrichment or depletion across all repeat superfamilies (Fig. 3f,g).

### COMPLETE-seq features improve classification performance of diagnostic models

**Fig. 4:**
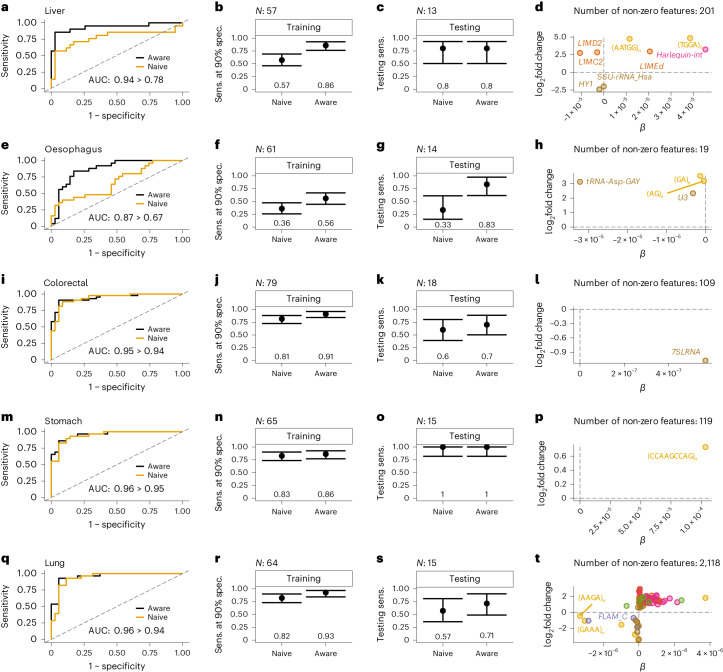
COMPLETE-seq features improve performance of diagnostic models. **a**,**e**,**i**,**m**,**q**, Receiver operating characteristic curves for the best repeat-aware model and the equivalent repeat-naive model for liver (**a**), oesophagus (**e**), colorectal (**i**), stomach (**m**), and lung (**q**) cancer. Diagonal lines represent a random classifier. AUC estimates are shown with the improved, repeat-aware AUC compared with the repeat-naive equivalent. **b**,**f**,**j**,**n**,**r**, Training sensitivity (sens.) at 90% specificity (spec.) for repeat-naive and repeat-aware models (95% confidence interval, binomial), for liver (**b**), oesophagus (**f**), colorectal (**j**), stomach (**n**), and lung (**r**) cancer, with values shown on the plot. **c**,**g**,**k**,**o**,**s**, Testing sensitivity calculated with the 90% specificity probability threshold identified in training (95% confidence interval, binomial), for liver (**c**), oesophagus (**g**), colorectal (**k**), stomach (**o**), and lung (**s**) cancer, with values shown on the plot. **d**,**h**,**l**,**p**,**t**, Comparison of model coefficient (*β*) to DESeq2 log_2_fold change for non-zero repeat features used in the repeat-aware model characterized in the respective row for liver (**d**), oesophagus (**h**), colorectal (**l**), stomach (**p**), and lung (**t**) cancer, with the total number of features shown. Horizontal and vertical lines drawn at 0.

To show proof of concept for diagnostic modelling using repeat-aware COMPLETE-seq analysis of cell-free RNA-seq data^32^, we trained logistic regression classifiers with tenfold cross-validation on training sets created for each cancer and healthy comparison (Fig. 4). To determine the utility of repeat-aware COMPLETE-seq features for disease classification, we trained models on repeat-naive and repeat-aware feature sets comprising DESeq2-normalized counts or biotype/repeat superfamily entropy for each cancer type. This resulted in eight feature sets comprising repeat-aware and repeat-naive counts, entropy and counts filtered by training set differential expression. Optimized repeat-aware models were compared with their repeat-naive counterparts, revealing repeat-driven increases in both area under the curve (AUC) (Fig. 4a,e,i,m,q) and training sensitivity for liver cancer (86% sensitivity) (Fig. 4b,c), oesophageal cancer (56% sensitivity) (Fig. 4f,g), colorectal cancer (91% sensitivity) (Fig. 4j,k), stomach cancer (86% sensitivity) (Fig. 4n,o) and lung cancer (93% sensitivity) (Fig. 4r,s) at 90% specificity.

Liver cancer, which showed a large repeat fraction (Extended Data Fig. 2a), had a corresponding dependence on repeat-aware features for classification, which resulted in a significant (*P* < 0.05) improvement in training sensitivity (Fig. 4a). Classification performance in the respective testing cohorts largely reflected the improvements seen in training, suggesting that our models have the potential to generalize to unseen data (Fig. 4c,g,k,o,s). Notably, we observed cancer-specific differences in repeat-aware feature dependence for disease classification. Stomach (Fig. 4p) and colorectal (Fig. 4l) cancer models each used one repeat-aware feature, liver (Fig. 4d) and oesophageal (Fig. 4h) cancer models used five and ten repeat-aware features, respectively, and the lung (Fig. 4t) cancer model used many repeat-aware features and the most overall features. For all five cancer models, repeat-aware features enhanced disease classification, highlighting the potential of COMPLETE-seq for highly sensitive and specific disease diagnosis.

## Discussion

Our study reveals the value and utility of broadly characterizing the cell-free RNA transcriptome using our repeat-aware COMPLETE-seq technology for RNA liquid biopsies. Although other studies have provided valuable insights into protein-coding cell-free RNA dynamics, we find that the vast non-coding and repeat-derived cell-free RNA transcriptome is a rich source of abundant and disease-specific RNA biomarkers. We show that repeat-derived cell-free RNAs, including simple repeat RNAs and TE RNAs transcribed from LINE, SINE and LTR elements, are cancer-specific RNAs that are normally present at low or undetectable levels in healthy individuals. By creating a custom, repeat-aware transcriptome annotation for cell-free RNA quantification that incorporates over 5 million repeat element insertions throughout the human genome, we show that repeat-derived cell-free RNAs are highly enriched in the plasma of patients with cancer, with each cancer type showing its own characteristic repeat-derived cell-free RNA signature. COMPLETE-seq also greatly reduces the number of features used for downstream analysis and disease classification from over 5 million repeat element insertions to ~15,000 aggregated repeat elements at the subfamily level. Notably, our repeat-aware approach achieves highly accurate disease classification by incorporating both protein-coding RNAs and non-coding RNAs, such as lncRNAs and repeat-derived RNAs.

Although cell-free RNA studies so far have focused on short-read RNA-seq technologies, we show that long-read RNA-seq technologies, such as single-molecule nanopore sequencing, provide additional information regarding the full length of cell-free RNAs. We see differences in cell-free RNA length (that is, bimodal SINE-derived cell-free RNAs) that may serve as additional disease-specific features to further improve disease classification via RNA liquid biopsy (that is, RNA fragmentomics). Moreover, we also show that COMPLETE-seq robustly characterizes repeat-derived cell-free RNAs in both poly(A)-selected and total RNA library preparation protocols. In both cell-free RNA-seq contexts, COMPLETE-seq analysis increases mapping rate significantly and provides a richer feature space that leverages highly abundant and disease-specific repeat-derived cell-free RNAs to improve classification performance.

COMPLETE-seq also provides systemic insights into disease pathogenesis, and opportunities to discover drug targets for diseases such as cancer. In addition, our RNA liquid-biopsy technology enables non-invasive, systemic monitoring of protein-coding and repeat-derived cell-free RNA responses to targeted therapies, such as KRAS inhibitors^36^, which induce treated cancer cells to preferentially release TE-derived cell-free RNAs in extracellular vesicles^9^. Given the preferential upregulation and secretion of TE-derived cell-free RNAs in response to mutant KRAS^8,9^, companion diagnostic tests developed using repeat-aware RNA liquid biopsy would enable the robust detection of repeat-derived cell-free RNA signatures of oncogenic RAS signalling.

To move towards clinical implementation of COMPLETE-seq, future studies will require the generation of larger and more diverse cell-free RNA transcriptomic datasets across additional early-stage cancer types to further improve diagnostic performance and to accurately deconvolve the cell-free RNA transcriptome to determine cancer tissue of origin. Moreover, multi-cancer early detection using COMPLETE-seq will also require larger prospective studies to evaluate repeat-aware classification performance in an asymptomatic population. These studies may enable the application of repeat-aware RNA liquid-biopsy technology for the early detection of cancer and, more generally, for precision health.

## Methods

### Cell-free RNA isolation from blood plasma

The ExoRNeasy kit (Qiagen) was used to isolate cell-free RNA from blood plasma of de-identified healthy controls (blood collected in K2EDTA tubes; Discovery Life Sciences) and patients with pancreatic cancer (blood collected in K2EDTA tubes; BioIVT). Samples were initially filtered through a 0.8 µm filter to remove any contaminants, such as buffy coat. Filtered plasma was then processed using the ExoRNeasy kit to isolate cell-free RNA according to manufacturer instructions.

### Library preparation for cell-free RNA-seq

Full-length cDNA was synthesized from cell-free RNA from pancreatic cancer and healthy control samples (Takara SMART-Seq HT kit). Size distribution of cell-free RNA and resulting cDNA were evaluated using an Agilent bioanalyser. Final libraries were made using the Illumina Nextera XT DNA Prep kit. These libraries were then sequenced (PE150) on an Illumina NextSeq 500.

Nanopore full-length cDNA libraries were prepared as described above, followed by manufacturer instructions for the Oxford Nanopore ligation kit LSK109. Sequencing for each library was performed on an Oxford Nanopore MinION device with R9.4 flow cells.

### RNA-seq quality control, alignment and quantification

RNA-seq reads (FASTQ) were trimmed with Trimmomatic^37^ (v.0.39), quality assessed using FastQC^38^ (v.0.11.9) and visualized using MultiQC^39^ (v.1.11). Quantification was performed using Salmon^40^ (v.1.9.0) with two separate transcriptome annotations:

1. The GENCODE consortium Hg38 reference annotation (v.39): 61,488 genes
2. A concatenation of the above reference and the Hg38 repeat element track available at the University of California Santa Cruz genome browser mySQL server genome-mysql.cse.ucsc.edu: ~5 million insertions

The following optional arguments were used:

–validateMappings, –gcBias, –seqBias, –recoverOrphans, –rangeFactorizationBins 4

to enable selective alignment, reduce sequence biases, rescue reads with unmapped pairs and improved quantification, respectively.

The repeat-aware annotation aggregation was performed much as the transcript-to-gene aggregation is performed for alignment-free quantification approaches such as Salmon. Briefly, we assembled a reference transcriptome that includes all annotated repeats in Hg38 and associated each individual repeat instance ‘transcript’ (*n* ≈ 5 million) with its subfamily ‘gene’ (*n* ≈ 15,000). Repeat instances were summated to the subfamily level.

Nanopore signals were base-called with guppy (v.5.0.7) and alignments were quantified using StringTie2 (ref. ^41^) with the ‘-L’ argument for long reads using the repeat-aware reference described above (number 2).

### Differential expression analysis

Salmon quantifications were loaded into R using tximeta (v.1.12.4)^42^ and converted to DESeq (v.1.34.0)^43,44^ objects where counts were aggregated to the gene level from transcripts. Count normalization was performed with DESeq2 and pairwise comparisons were calculated with the following models:

Internal cohort: ~age + gender + input_volume + condition

External cohort: ~age + gender + condition

Significant differential expression was considered at adjusted *P* values (*P*_adj_) of <0.01.

### Unsupervised analysis

Using the gene-level, variance-stabilized counts computed above, principal component analysis was performed with the prcomp from the R stats (v.4.1.3) package on a count matrix filtered to include only genes with non-zero s.d. across the samples using the following optional arguments: centre = T scale. = T rank. = 50 to calculate the 50 principal components from centred, scaled counts.

Pearson correlation was calculated using the cor function from the R stats (v.4.1.3) package. *K*-means clustering was calculated and plotted with the ComplexHeatmap package in R using, where appropriate, Pearson correlation values (described above) or expression *Z* scores calculated with the scale function in R with ‘centre = T’ to centre our scaled values at zero.

### Cell-free RNA length analysis

lncRNA, protein-coding and TE reference sequences were retrieved as described above. Sequences were extracted from Hg38 to create the biotype reference genomes used in the length analyses. Nanopore reads were aligned to Hg38 using Minimap2. To determine alignments in genomic regions with overlapping annotations, the length of the aligned fragment was compared with the lengths of the overlapping repeats. The annotation with the closest length to that of the fragment was chosen as the correct alignment. Fragment length was extracted using the PySam (v.0.15.4) template length.

### Modelling and statistical analysis

#### Feature engineering

DESeq2-normalized counts of features with a non-zero s.d. were used directly in all cases except where they were used to calculate Shannon entropy. Entropy (*H*) was calculated on a per-sample basis for each biotype or subfamily as:

$${H}_{{\mathrm{biotype}}}=-\mathop{\sum }\limits_{i}^{n}{p}_{i}{{\rm{log }}}_{2}({p}_{i})$$

where *p*_*i*_ represents the fractional contribution of a given feature *i* (total of *n*) belonging to the biotype of interest to the total biotype abundance.

For classification as described below, eight feature sets belonging to three categories were used as input matrices for model training:

1. Total: repeat-naive, repeat-aware and repeat-alone features
2. Differential expression: repeat-naive, repeat-aware and repeat-alone differentially expressed features (calculated on training set, excluding test set)
3. Entropy: TE clade entropy and TE clade entropy plus repeat-naive features

#### Classification and performance evaluation

Each disease cohort was paired with healthy samples and split into stratified training (80%) and testing (20%) subsets. Training splits were used to optimize logistic regression models by performing tenfold cross-validated classification using elastic net penalty values (*ɑ*) from zero (lasso) to one (ridge) to optimize feature selection via the regularization parameter (*λ*), producing a final model trained on the entire training split with selected features. Top-performing models were identified by training sensitivity at 90% specificity, which was determined by calculating the probability threshold that achieved ~90% specificity and AUC.

When feature sets including differentially expressed genes were used, differential expression was calculated using only the training split and excluding testing samples. Model performance was finally evaluated on held-out test data by generating prediction probabilities on the test split samples and classifying based on the 90% specificity probability threshold defined in training. Features with non-zero coefficients (*β*) in the final models were identified to determine total feature size. Confidence intervals for sensitivity were estimated as binomial confidence intervals based on the successful observations and the total training/testing cohort. All modelling was performed with the cv.glmnet function from the glmnet package and custom code written in R.

#### Statistical analysis

Unless otherwise stated, comparison of means was performed with a two-sided, unpaired Wilcoxon rank-sum test. Where paired tests are used, lines are drawn to connect the dependent observations. When represented using symbolic ranks (*), statistical significance is defined as follows: non-significant *P* > 0.05, *P ≤ 0.05, **P ≤ 0.01, ***P ≤ 0.001 and ****P ≤ 0.0001.

### Reporting summary

Further information on research design is available in the Nature Portfolio Reporting Summary linked to this article.

### Supplementary information

Reporting Summary

Supplementary Information Metadata describing critical clinical features of the 32-member cohort generated for this study, and available at https://www.ncbi.nlm.nih.gov/geo/query/acc.cgi?acc=GSE138651.

## Data Availability

The main data supporting the results in this study are available within the article and its Supplementary Information. RNA-seq data are available at the NCBI Gene Expression Omnibus repository, under accession number GSE136651. Publicly available data used in this study are available at the NCBI Gene Expression Omnibus repository, under accession number GSE174302. All data generated in this study, including source data for the figures, are available from the corresponding author on reasonable request.

## Code availability

Custom code used in this study is available on GitHub at https://github.com/rreggiar/exRNA_disease_biomarkers.
