# Supplementary material for: Profiling of repetitive RNA sequences in the blood plasma of patients with cancer
Source: Nat Biomed Eng. 2023 Aug 31;7(12):1627–35. doi: 10.1038/s41551-023-01081-7 (PMC10727983; doi:10.1038/s41551-023-01081-7)
Supplement: Supplementary file 1 — Reporting Summary [file 41551_2023_1081_MOESM1_ESM.pdf]

nature portfolio

Corresponding author(s): Daniel H. Kim

Last updated by author(s): Jul 6, 2023

Reporting Summary

Nature Portfolio wishes to improve the reproducibility of the work that we publish. This form provides structure for consistency and transparency in reporting. For further information on Nature Portfolio policies, see our Editorial Policies and the Editorial Policy Checklist.

Statistics

For all statistical analyses, confirm that the following items are present in the figure legend, table legend, main text, or Methods section.

n/a	Confirmed	
☐	☒	The exact sample size (*n*) for each experimental group/condition, given as a discrete number and unit of measurement
☐	☒	A statement on whether measurements were taken from distinct samples or whether the same sample was measured repeatedly
☐	☒	The statistical test(s) used AND whether they are one- or two-sided<br>*Only common tests should be described solely by name; describe more complex techniques in the Methods section.*
☐	☒	A description of all covariates tested
☐	☒	A description of any assumptions or corrections, such as tests of normality and adjustment for multiple comparisons
☐	☒	A full description of the statistical parameters including central tendency (e.g. means) or other basic estimates (e.g. regression coefficient) AND variation (e.g. standard deviation) or associated estimates of uncertainty (e.g. confidence intervals)
☐	☒	For null hypothesis testing, the test statistic (e.g. F, t, r) with confidence intervals, effect sizes, degrees of freedom and P value noted<br>*Give P values as exact values whenever suitable.*
☒	☐	For Bayesian analysis, information on the choice of priors and Markov chain Monte Carlo settings
☒	☐	For hierarchical and complex designs, identification of the appropriate level for tests and full reporting of outcomes
☐	☒	Estimates of effect sizes (e.g. Cohen's *d*, Pearson's *r*), indicating how they were calculated

Our web collection on statistics for biologists contains articles on many of the points above.

Software and code

Policy information about availability of computer code

Data collection	RNA-sequencing data were generated using an Illumina NextSeq 500, and base-called with up-to-date Illumina Real Time Analysis software.
Data analysis	FastQC (0.39), MultiQC (1.11), Salmon (1.6.0) were used for QA/QC, alignment, and quantification of RNA-sequencing data, respectively.<br>The R programming language (4.3.1), and relevant packages, were used to analyse and visualize the data.

For manuscripts utilizing custom algorithms or software that are central to the research but not yet described in published literature, software must be made available to editors and reviewers. We strongly encourage code deposition in a community repository (e.g. GitHub). See the Nature Portfolio guidelines for submitting code & software for further information.

Data

Policy information about availability of data

All manuscripts must include a data availability statement. This statement should provide the following information, where applicable:

- Accession codes, unique identifiers, or web links for publicly available datasets
- A description of any restrictions on data availability
- For clinical datasets or third party data, please ensure that the statement adheres to our policy

The main data supporting the results in this study are available within the paper and its Supplementary Information. RNA-seq data are available at the NCBI Gene Expression Omnibus repository, under accession number GSE136651. Publicly available data used in this study are available at the NCBI Gene Expression Omnibus

repository, under accession number GSE174302. All data generated in this study, including source data for the figures, are available from the corresponding author on reasonable request.

Human research participants

Policy information about studies involving human research participants and Sex and Gender in Research.

Reporting on sex and gender	Sex is reported as a covariate, where appropriate.
Population characteristics	Diagnosis (healthy, pancreatic ductal adenocarcinoma), age, sex and stage of disease.
Recruitment	Samples were purchased from the clinical research organizations BioIVT and Discovery.
Ethics oversight	Because we purchased the de-identified samples, we received IRB exemption (the study is not considered to be research involving human participants).

Note that full information on the approval of the study protocol must also be provided in the manuscript.

Field-specific reporting

Please select the one below that is the best fit for your research. If you are not sure, read the appropriate sections before making your selection.

☒ Life sciences ☐ Behavioural & social sciences ☐ Ecological, evolutionary & environmental sciences

For a reference copy of the document with all sections, see nature.com/documents/nr-reporting-summary-flat.pdf

Life sciences study design

All studies must disclose on these points even when the disclosure is negative.

Sample size	32 total (N = 10 Pancreatic Cancer, N = 22 Normal healthy donors) blood-plasma samples were used for the study, to enable preliminary observations regarding the information available in the assay and the analytical approach. Publicly available (N = 295) data were used to perform diagnostic modelling.
Data exclusions	No data were excluded.
Replication	Each of the 32 samples was sequenced once.
Randomization	All RNA-sequencing data prepared from human blood plasma were analysed equally.
Blinding	All sample identities were known throughout the study.

Reporting for specific materials, systems and methods

We require information from authors about some types of materials, experimental systems and methods used in many studies. Here, indicate whether each material, system or method listed is relevant to your study. If you are not sure if a list item applies to your research, read the appropriate section before selecting a response.

Materials & experimental systems

n/a	Involved in the study	
☒	☐	Antibodies
☒	☐	Eukaryotic cell lines
☒	☐	Palaeontology and archaeology
☒	☐	Animals and other organisms
☒	☐	Clinical data
☒	☐	Dual use research of concern

Methods

n/a	Involved in the study	
☒	☐	ChIP-seq
☒	☐	Flow cytometry
☒	☐	MRI-based neuroimaging
